# Alteration of Energy Substrates and ROS Production in Diabetic Cardiomyopathy

**DOI:** 10.1155/2013/461967

**Published:** 2013-10-31

**Authors:** O. Lorenzo, E. Ramírez, B. Picatoste, J. Egido, J. Tuñón

**Affiliations:** ^1^IIS-Fundación Jiménez Díaz, Autónoma University, Avenue Reyes Católicos 2, 28040 Madrid, Spain; ^2^Spanish Biomedical Research Centre in Diabetes and Associated Metabolic Disorders (CIBERDEM) Network, 08017 Barcelona, Spain

## Abstract

Diabetic cardiomyopathy is initiated by alterations in energy substrates. Despite excess of plasma glucose and lipids, the diabetic heart almost exclusively depends on fatty acid degradation. Glycolytic enzymes and transporters are impaired by fatty acid metabolism, leading to accumulation of glucose derivatives. However, fatty acid oxidation yields lower ATP production per mole of oxygen than glucose, causing mitochondrial uncoupling and decreased energy efficiency. In addition, the oxidation of fatty acids can saturate and cause their deposition in the cytosol, where they deviate to induce toxic metabolites or gene expression by nuclear-receptor interaction. Hyperglycemia, the fatty acid oxidation pathway, and the cytosolic storage of fatty acid and glucose/fatty acid derivatives are major inducers of reactive oxygen species. However, the presence of these species can be essential for physiological responses in the diabetic myocardium.

## 1. ROS Balance in the Diabetic Cardiac Cell

The number of mitochondria a cell contains depends on how much energy the cell needs to produce. In particular, cardiac cells comprise a large number of mitochondria for continuous aerobic respiration via oxidative phosphorylation [1]. These organelles are responsible for 90% of the energy that cells need to function; however, mitochondria are also the primary source of reactive oxygen species (ROS) in mammalian cells [2]. ROS include hydrogen peroxide (H_2_O_2_), oxygen ions (O_2_^−^), and hydroxyl and superoxide radicals (∙OH and ∙O_2_^−^, resp.). H_2_O_2_ is a weak oxidizing agent. However, by a Fenton reaction with iron, H_2_O_2_ yields ∙OH, the most oxidizing of all cellular ROS. Moreover, ∙O_2_^−^ can spontaneously combine with NO radicals (∙NO) produced by inducible nitric oxide synthase (iNOS) to form peroxynitrite (ONOO^−^), which rapidly degrades to highly oxidant reactive nitrogen species such as nitronium ion [3]. ROS are continuously produced during many physiological processes in the cytosol and mitochondria of cardiac cells mainly by four enzyme systems: the mitochondrial respiratory chain, NADPH oxidase (NOX), xanthine oxidase, and endothelial NOS (eNOS) [4]. However, ROS production can be in equilibrium with the antioxidant capacity of the cell. In this sense, there are some natural detoxification molecules that reduce or scavenge ROS: enzymatic molecules, such as superoxide dismutase (SOD), hemoxigenase-1 (HO-1), reduced glutathione (GSH), glutathione peroxidase (GPox), and catalase, as well as other nonenzymatic antioxidants, such as transferrin, ferritin, and vitamins A, E, and C [5]. All of these antioxidant systems can decrease the potency of particular reactive species or render them completely harmless. Unfortunately, this antioxidant defense may not counteract the prooxidant effect developed in diabetic cardiomyopathy (DCM), a unique entity by which diabetes affects heart muscle independently of any vascular disease or hypertension. In this sense, Akhileshwar et al. demonstrated that while SOD and GPox activities were increased in the diabetic rat heart, catalase and GSH were ameliorated [6]. In this regard, by exogenous antioxidant stimulation or ROS neutralization (i.e., by modulation of mitochondrial activity), scavenging (i.e., by interruption of ROS overproduction), or interference with ROS-induced alterations (i.e., by regulation of ROS mediators), various therapies have demonstrated an improvement in DCM pathology [3, 5, 7].

## 2. ROS and Cell Signalling in DCM

ROS can be ideal signalling molecules due to the high complexity and control by which they are synthesized and degraded [8]. ROS contain one or more unpaired electrons, making them extremely susceptible to interaction with biological molecules such as DNA, lipids, carbohydrates, and proteins. In the DNA, ROS, especially, H_2_O_2_, can interact with both the bases and the sugar residues of the nucleic acid sequence. ROS-related oxidation of DNA is one of the main causes of mutations, such as nonbulky (8-oxoguanine and formamidopyrimidine) and bulky (cyclopurine and etheno adducts) base modifications, abasic sites, nonconventional single-strand breaks, protein-DNA adducts, and intra- or interstrand DNA crosslinks [9]. Lipids, mostly polyunsaturated fatty acids (FA), can be oxidized since they contain multiple double bonds between which lie methylene bridges that possess especially reactive hydrogens. The resultant FA radical is unstable and reacts readily with molecular oxygen, thereby creating a peroxyl-FA radical. This radical reacts with another FA, yielding lipid peroxide, which can also cause extremely oxidant products such as unsaturated aldehydes (i.e., 4-hydroxy-2-nonenal (4-HNE)) and intensify lipid peroxidation [10]. Moreover, ROS interact with carbohydrates, and the subsequent oxidized carbohydrates can glycate proteins (by Maillard reactions) and form advanced glycation products (AGEs). AGEs may be even more reactive than the initial sugars they were constituted from. The mechanism by which AGEs induce damage is through a process called crosslinking (proteins can be covalently bonded forming chains), which causes intracellular damage and apoptosis [11]. Oxidations in proteins are described as posttranslational modifications on methionine/cysteine residues of several oxidoreductases, kinases, proteases, and transcription factors, thus affecting downstream enzymatic activity, membrane structure, and gene signalling [12]. In this manner, the heart offers “redox-sensor” molecules that are highly susceptible to the oxidative diabetic environment. Importantly, ROS may interact on producer and neighboring cells during DCM.

### 2.1. Extracellular ROS Signalling

**Figure 1:**
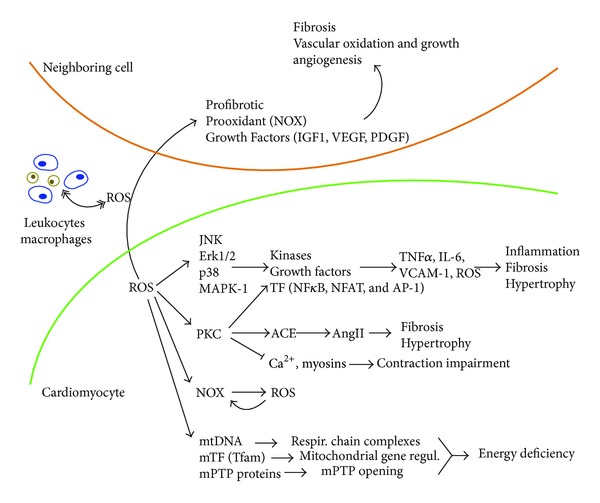
Paracrine and autocrine effects of ROS in DCM. ROS may be secreted from cardiomyocytes to induce responses in remote (inflammatory) and neighboring (vascular, endothelial, and fibroblast) cells. Within the cardiomyocytes, ROS could also influence different cytosolic (MAPK, PKC, and NOX) and mitochondrial mediators. mtDNA, mTF, and mPTP mean mitochondrial DNA, mitochondrial transcription factors, and mitochondrial permeability transition pores, respectively.

Once formed, ROS can be released from cardiac cells and act in recruiting the first wave of inflammatory cells to the injury [13]. Several ROS, in particular, H_2_O_2_, are membrane permeable and are, therefore, potential candidates to signal other cells within the inflammatory milieu, as occurs in DCM (Figure 1). Particularly, leukocytes can be chemoattracted by H_2_O_2_ gradients [14], and T-cells activate and proliferate following a ROS increase [15]. Also, elevation of ROS in cardiac stem cells triggers a paracrine secretion of growth factors such as insulin growth factor-1 (IGF-1) and stromal-derived factor-1 (SDF-1) into conditioned media [16]. Rapid depolarization of atrial myocytes increases secretion of ROS, which stimulate the profibrotic expression in cocultured fibroblasts [17]. Moreover, ∙O_2_^−^ may play a paracrine role in the regulation of vascular function. It has been showed that ∙O_2_^−^ induced cardiac NOX activity and expression of the NOX subunit gp91phox in endothelial cells, leading to coronary endothelial dysfunction [18]. ROS also play a crucial role in angiogenesis. Vascular repair and expression of angiogenic genes including vascular endothelial growth factor (VEGF), fibroblast growth factor (FGF), and platelet-derived growth factor (PDGF) were upregulated after cardiac secretion of ROS [19]. Thus, as a stress-response mechanism, the diabetic myocardium could release ROS to activate inflammation and other processes (e.g., angiogenesis) in remote and neighboring areas.

### 2.2. Intracellular ROS Signalling

Cytosolic ROS are mainly produced by NOX in the heart [20]. The redox state plays a central role in multiple signalling cascades such as mitogen-activated kinases (MAPKs) (Figure 1). ROS promote the phosphorylation and activation of c-Jun NH2-terminal kinases (JNKs), extracellular signal-regulated kinase-1/-2 (ERK1/2), p38-MAPKs, and the big MAPK-1 [21, 22]. In this sense, the lack of p38 prevented ROS-induced apoptosis and remodeling in DCM [23]. In addition, once activated, MAPKs can transactivate several other kinases, growth factors, and transcription factors accompanied by the related gene expression [24]. Nuclear factor *κ*B (NF*κ*B), activating protein-1 (AP-1), early growth response gene-1 (Egr-1), surfactant protein-1 (SP-1), and E26 transformation specific-1 (Ets-1) are ROS-induced transcription factors [25]. NF*κ*B is a transcription factor that, as a part of a stress response, binds promoters of a plethora of cytokines, chemokines, adhesion molecules, and enzymes involved in secondary inflammatory mediation. Interestingly, diabetic cardiac biopsies showed higher NF*κ*B levels and subsequent NF*κ*B-target genes, like TNF*α* [26]. Some data also provide evidence that protein kinase-C- (PKC-) dependent redox Signalling affects cardiac function [27]. The increase of PKC in diabetic hearts also elevates the expression of angiotensin-II-converting enzyme (ACE) and myosin heavy chain and interferes with proteins involved in cardiac excitation-contraction coupling [28, 29]. Upon electrical stimulation, Ca^2+^ is released from the sarcoplasmic reticulum to bind actin filaments, allowing cross-bridges between actin and myosin. Ca^2+^ is a reuptake for a new contractile cycle via Ca^2+^-ATPase-2a. Moreover, ROS slow down sarcoplasmic Ca^2+^ uptake and decrease L-type Ca^2+^ flow amplitude, damaging cell contraction [30]. Under G-protein-dependent PLC activation and 1,2-diacylglycerol (DAG) release, PKC is activated and stimulates transcription factors such as nuclear factor of activated T-cells (NFAT) and NF*κ*B [31]. Interestingly, low levels of cytosolic ROS stimulate NF*κ*B, while high levels of cytosolic and nuclear ROS inactivate NF*κ*B-DNA binding [12]. Finally, ROS have the ability to positively increase NOX activity and thus further induce ∙O_2_^−^ production [32]. 

Mitochondria are the major source of ROS in the cell. Mitochondrial ROS are synthesized as a byproduct of oxygen metabolism by the respiratory chain (from complexes I and III, in the inner mitochondrial membrane) and by nonrespiratory enzymes (i.e., monoamine oxidase, cytochrome B5, or pyruvate dehydrogenase complex, in the outer membrane) [33]. However, the bulk of mitochondrial ROS generation occurs at the electron transport chain [2]. Moreover, ROS can affect mitochondrial DNA (mtDNA), which is a closed-circular double-stranded DNA molecule of *∼*16.5 kb encoding for components of the mitochondrial respiratory machinery (13 polypeptides, 22 tRNAs, and 2 rRNAs) [34]. ROS can also mediate the opening of mitochondrial permeability transition pores (mPTP) by membrane lipid peroxidation, stimulating increased inner membrane permeability, Ca^2+^ release, and ATP depletion in the diabetic myocardium [35, 36]. In addition, the activity of the mitochondrial transcription factors A (Tfam), involved in mitochondria biogenesis, can also be regulated by ROS [37, 38]. 

Therefore, both cytosolic and mitochondrial ROS could regulate the activity and expression of multiple mediators in the diabetic myocardium.

## 3. Diabetic Heart and ROS Generation

**Figure 2:**
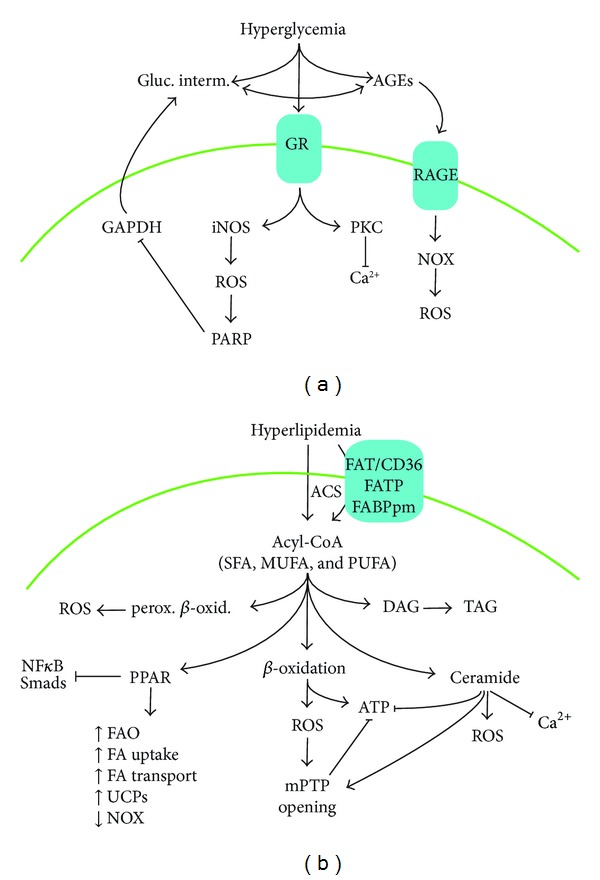
Hyperglycemia, hyperlipidemia, and myocardial ROS production. In diabetes, both the excess of plasma glucose (a) that cannot be properly assimilated by the tissues and the abundance of plasma lipids (b) may induce oxidative stress in the heart by different mediators. UCPs, uncoupling proteins.

Most extracellular insults, such as concentration of energetic substrates, can cause an increase of ROS, eventually surpassing tissue demand [22]. Oxidative stress is defined as an oversupply of ROS relative to the antioxidant defense. The destructive role of ROS is attributed to their deforming properties on biological molecules and consequent influence on cell fate and phenotype [13]. In diabetes, alteration of energy substrates (i.e., excess of plasma glucose and lipids) can directly or indirectly induce ROS formation in the myocardium. DCM begins with a disturbance in the glucose metabolism that provokes hyperglycemia. A feature common to all cell types that are damaged by hyperglycemia is an increased production of AGEs and ROS [39, 40]. High concentration of glucose triggers AGEs synthesis, in particular, glycated extra matrix proteins (e.g., collagens). AGEs bind specific receptors (RAGE) to activate NOX and to release ROS [41] (Figure 2(a)). PKC can also be stimulated by AGEs to phosphorylate proteins involved in Ca^2+^ handling and cardiomyocyte contraction [42, 43]. In addition, acute exposure to high glucose elevates iNOS gene expression and subsequent ∙NO, ∙O_2_^−^, and ONOO^−^ [44], which in turn stimulate poly-ADP ribose-polymerase-1 enzyme (PARP) as a compensatory antioxidant mechanism. In this sense, hyperglycemia caused upregulation of extracellular matrix proteins and cardiomyocyte hypertrophy and increased oxidative stress in diabetic hearts, and these alterations were not found in the PARP(−/−) mice and diabetic rats treated with a PARP inhibitor [45]. However, PARP inhibits the glycolytic enzyme glyceraldehyde 3-phosphate dehydrogenase (GAPDH), and thus it induces glycolytic intermediate formation and, again, AGEs [4, 45] (Figure 2(a)). High glucose concentration increases the flux through the hexosamine biosynthetic pathway and elevates N-acetylglucosamine, which has been associated with insulin resistance and ROS production [46]. Furthermore, as a consequence of hyperglycemia, insulin resistance and hyperinsulinemia elicit an elevation of plasma lipids in diabetes. Obesity is also frequently present in the diabetic patient, and lipolysis of adipose tissue is incompletely suppressed, causing increased release of FA [47]. Several studies have reported that hyperlipidemia induces overproduction of mitochondrial ROS in diabetic patients [48].

The diabetic heart prefers FA as an energy substrate [49]. In type II diabetic patients, a twofold increase in cardiac palmitate oxidation and a 30%–40% decrease in glucose oxidation have been described [50]. Serum FA composition (cholesterol esters, phospholipids, and triacylglycerols (TAG)) is roughly reproduced by the FA composition of the diet [51]. However, plasma FA is also affected by other factors such as gender and physical activity, and some FA can be generated by *de novo* synthesis [52]. Most of the plasma lipids are TAG, containing unbranched monocarboxylic FA with at least sixteen carbons (long-chain FA). Depending on the presence of double bonds, FA are classified as saturated (SFA, without double bonds), monounsaturated (MUFA, with one double bond), and polyunsaturated (PUFA, with at least two double bonds). MUFA/PUFA and SFA can be converted by desaturases/elongases in the endoplasmic reticulum (ER) [53]. Among PUFA, linoleic and alpha-linolenic acids are named “essential FA” (only obtained from the diet), and their derivatives, such as arachidonic acid, play pivotal roles in cardiac FA metabolism (see below). Importantly, excessive levels of dietary fat or higher content of SFA versus MUFA/PUFA has been implicated in the onset and progression of several chronic diseases, including diabetes and obesity [54]. Cardiac cells can take up circulating FA (as albumin associated with or coupled in lipoproteins) by simple diffusion across the plasma membrane or by FA transporters (i.e., FAT/CD36, FABPpm, and FATP1, -4, and -6 [55] (Figure 2(b)). In particular, FAT/CD36 is responsible for up to 60% of the FA uptake in the heart and, like the glucose transporter (Glut4), can traffic between endosomes and plasma membrane following stimulation (insulin, muscle contraction, and AMPK activation) [56]. Once in the cells, membrane-associated acyl-CoA synthetases generate acyl-CoAs (from FA and coenzyme-A) that may provide substrates for membrane structure, energy metabolism, and signalling molecules. Nonetheless, an overload of acyl-CoAs can saturate FA delivery and deviate to undesirable outcomes. In the diabetic myocardia, both lipid degradation and accumulation cause cytosolic and mainly mitochondrial ROS release [57]. However, lipid may also decrease the oxidation state by regulating specific gene expression.

### 3.1. *β*-Oxidation and ROS Production

Acyl-CoAs are rapidly metabolized by peroxisomal and, mainly, mitochondrial *β*-oxidation, leaving intracellular acyl-CoA concentration at very low levels (<10 *µ*M) [58]. The transport of acyl-CoA into the mitochondria is accomplished by an acylcarnitine intermediate, which is generated by the carnitine palmitoyltransferase (CPT) system at the mitochondrial outer and inner membranes. Once inside the mitochondrion, acyl-CoA is the substrate for the *β*-oxidation machinery in which a carbon at the *β*-position is oxidized in every round of the cycle (Figure 2(b)). Several flavin adenine dinucleotide- (FAD-) dependent acyl-CoA dehydrogenases and a mitochondrial trifunctional enzyme (with enoyl-CoA hydratase, 3-hydroxyacyl-CoA dehydrogenase, and thiolase activities) compose this pathway. Each round of *β*-oxidation produces one mole of each FADH_2_, NADH, and acetyl-CoA. The latter enters into the tricarboxylic-acid cycle to oxidize CO_2_ and generate three moles of NADH and one mole of each FADH_2_ and ATP. Then, all generated NADH and FADH_2_ are used within the respiratory pathway for the production of more ATP via oxidative phosphorylation. In this process, ROS, mostly, ∙O_2_^−^, are constitutively produced when both NADH and FADH_2_ directly react with oxygen [59]. In the diabetic myocardium, increased acyl-CoA and *β*-oxidation trigger the delivery of reducing equivalents (NADH and FADH_2_) to the electron transport chain and the generation of ROS [60, 61] (Figure 2(b)). Also, the increase in FA metabolism in the diabetic heart has been associated with an increased expression of mitochondrial uncoupling proteins (UCP). In particular, FA can induce UCP3 via PPAR*α* activation [62]. However, despite the fact that mitochondrial uncoupling serves to limit the production of ROS (by reducing proton gradient), uncoupling can be deleterious since it lowers the production of ATP, thereby making the heart less efficient. In addition, the excess of acyl-CoA also activates the peroxisomal *β*-oxidation in DCM. This process plays an important role in overall FA metabolism of the diabetic heart, mainly for very long-chain acyl-CoA and acyl-CoA derivatives. However, first oxidation steps in peroxisomes are not coupled to energy production, implying an extra source of ROS [60].

### 3.2. Lipid Derivatives and ROS Generation

In the diabetic heart, an overloaded *β*-oxidation can saturate the mitochondria, leading to the accumulation of acyl-CoA in the cytosol. The deposition of acyl-CoA in nonadipose tissues is called steatosis. Myocardial steatosis may lead to cell dysfunction and death. In addition to recycling to TAG by esterification (Figure 2(b)), acyl-CoAs can be rapidly assimilated into complexes such as phospholipids and mainly sphingolipids (e.g., ceramides and glycosylceramides) at the ER [48]. Especially, SFA, but not PUFA or MUFA, are converted to sphingolipids by the serine-palmitoyl-CoA transferase activity. Sphingolipids possess both signalling and structural properties, and some of them, like sphingosine-1 phosphate, are antioxidant and cardioprotective [63]. However, others like ceramides may cause cardiac failure. In this sense, in various rodent models of diabetes, increased myocardial ROS and ceramide content have been associated with cardiac dysfunction [64]. Ceramides also promoted apoptosis, insulin resistance, autophagy, and inflammation [63] and acted also directly on mitochondria to inhibit complex III and generate ROS and inflammation [65] (Figure 2(b)). Ceramides activate proapoptotic protein phosphatase-1 or -2A (PP1 or PP2A) and cathepsin D [66]. In addition, ceramides have been showed to inhibit Ca^2+^ release and cell contraction in the diabetic myocardium [67]. Methods to reduce cardiac ceramide synthesis, including the decrease of FA uptake and conversion of FA to nontoxic TAG, have demonstrated beneficial effects [66].

### 3.3. Lipid Signalling and ROS Control

Lipids have also been recognized as function-like nuclear signalling molecules. Acyl-CoAs can be transported to the nucleus by FA-binding proteins [68]. SFA, MUFA, or PUFA and, in this case, arachidonic acid metabolites such as eicosanoids (prostaglandins, leukotrienes, thromboxanes, and lipoxins) can regulate lipid homeostasis and oxidation by interacting with nuclear receptors. Of the five nuclear-receptor families reported to bind acyl-CoAs, only peroxisome proliferator-activated receptors (PPARs) are well accepted as FA-regulated nuclear receptors [69]. Two isotypes of PPARs are mostly expressed in the myocardium (PPAR*α* and PPAR*β*/*δ*). Based on their structural and chain composition, acyl-CoAs will show different affinity for the PPAR subtypes [53]. PPARs heterodimerize with retinoid-X receptors (RXR) and mediate transcriptional activation by binding to specific DNA promoter sequences in target genes. Activation of PPAR*α* and PPAR*β*/*δ* is linked to the upregulation of several genes involved in FA uptake, binding, and *β*-oxidation, and, thus, PPARs promote ROS generation in both peroxisomes and mitochondria [70, 71] (Figure 2(b)). However, pharmaceutical stimulation of PPAR*α* and PPAR*β*/*δ* induces an increase of *β*-oxidation and reduction of lipid accumulation and cardiac dysfunction. Intriguingly, in contrast with PPAR*β*/*δ*-transgenic mice, PPAR*α*-overexpressing mice develop cardiac hypertrophy and dysfunction, associated with myocardial lipid accumulation and high FA uptake and utilization rates [72, 73]. This pathology has been linked to the excess of ROS production. PPAR*α* activation can also decrease glucose utilization by increasing the expression of pyruvate dehydrogenase kinase-4 (PDK4), and the agonism of PPAR*α* elevates cardiac ceramide levels and leads to cardiac dysfunction [74]. In addition, exogenous simulation of PPAR*β*/*δ* could also prevent the expression of NOX subunits (p22(phox) and p47(phox)) and, resulting elevation of NOX activity and ∙O_2_^−^ production [75]. In this sense, some PPAR-acyl-CoA complexes can also regulate gene expression of prooxidant signals, such as NF*κ*B, in a DNA-independent way (Figure 2(b)). PPARs sequestrate NF*κ*B coactivators and/or stimulate I*κ*B*α* (the NF*κ*B inhibitory subunit) to prevent NF*κ*B translocation to the nucleus and later oxidation/inflammation [76, 77]. Altogether, the use of exogenous ligands for specific PPAR isoforms may overexpress FA-metabolic genes but may also compensate the oxidative stress by regulation of prooxidant genes and NF*κ*B. 

## 4. Deleterious Effects of ROS in DCM

**Figure 3:**
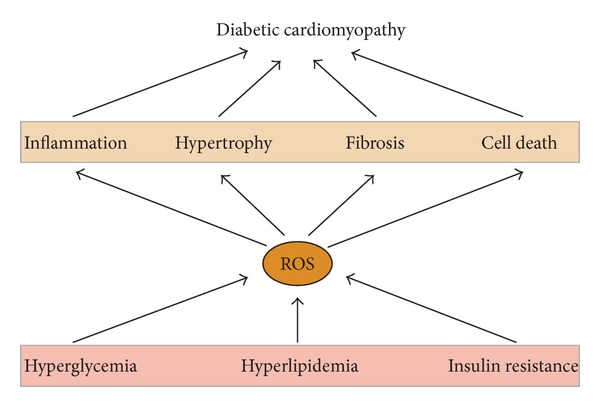
Pathological responses mediated by ROS in the diabetic heart. ROS are involved in the proinflammatory, -hypertrophic, and -fibrotic as well as cell-death processes developed in the diabetic myocardium.

In the initial step of DCM, patients exhibit diastolic heart failure with normal ejection fraction often associated with hypertrophy. Later, patients present systolic and diastolic heart failure with dilatation and reduced ejection fraction (stage 2). Patients with additional myocardial inflammation (and/or microvascular disease) are classified as DCM stage 3. Finally, patients with ischemia, infarction, and remodelling are enclosed in DCM stage 4 [78]. Importantly, the underlying cellular processes including inflammation and hypertrophy, myocyte apoptosis and necrosis, and deposition of extracellular matrix have a direct correlation with the oxidative state of the cardiac cell (Figure 3). 

### 4.1. ROS and Cardiac Inflammation

Obesity and type II diabetes are characterized by a state of low-grade inflammation [79]. The hypoxic and prooxidant status of the diabetic heart is usually characterized by elevated production and release of proinflammatory cytokines (such as TNF*α* and IL-6), which activate the NF*κ*B signalling, macrophage and T-cell recruitment, and leukocyte cardiac infiltration [80, 81]. Epicardial adipose tissue, which is increased in obese patients, may interact with myocardium through secretion of proinflammatory cytokines [82]. ROS also trigger PARP, which in turn activates NF*κ*B and proinflammatory genes [4]. Some data also provide evidence that p38- and PKC-dependent redox signalling are involved in cardiac inflammation [83, 84]. Importantly, recruited inflammatory cells can also release TNF*α* and IL-6, enhancing inflammation. In this regard, ROS mediate upregulation of MCP-1, and this chemokine also stimulates ROS production [85, 86]. MMP9, a metalloproteinase regulated by ROS, also increases inflammation by stimulation of proinflammatory TNF*α* [87]. Of interest, specific acyl-CoAs like SFA may directly bind and activate proinflammatory Toll-like receptor-4 (TLR4) [88]. In this regard, TLR4-deficient diabetic mouse hearts showed lower TAG accumulation and improved cardiac function [89]. On the other hand, hyperglycemia can induce epigenetic modifications on histones that increase the expression of p65 NF*κ*B subunit and consequent inflammation [90]. In this regard, exposure to high glucose also caused overexpression of IL-6 and MCP-1 through decreased histone-3 methylation at the cytokine promoter [91]. 

### 4.2. ROS and Cardiac Hypertrophy

The development of cardiac hypertrophy may involve an increase in mitochondrial ROS and mitochondrial DNA damage [92]. However, hypertrophy may also be a consequence of distinct stimulated sources of ROS, including mechanical strain, and the activation of G-protein-coupled receptors, receptor tyrosine kinases, and natriuretic peptides [93]. Ligands for G-protein-coupled receptors (e.g., angiotensin-II, endothelin-1, and adrenaline) induce hypertrophy by NOX-ROS-dependent mediators such as Erk1/2 and NF*κ*B [94]. Other signalling pathways involved in hypertrophy are potentially subject to redox regulation, such as PI3K/Akt, PKA, JAK-STAT, and NFAT [95]. ROS also activate MAPK and MMPs, which contribute to cardiac growth and hypertrophic responses [24, 96]. Angiotensin-II, a key effector of the renin-angiotensin-aldosterone system (RAAS) involved in the progression of myocardial hypertrophy to heart failure in diabetes, is also activated by ROS [97]. Finally, induction of hypertrophic markers such as brain natriuretic peptide (BNP) may depend on ROS-activated NF*κ*B [98]. 

### 4.3. ROS and Cardiac Fibrosis

In DCM, the development of fibrosis involves not only fibroblasts but also cardiomyocytes and endothelial and inflammatory cells. Interstitial fibrosis is a key component in cardiac dysfunction typically linked to RAAS activation in a proinflammatory atmosphere, with transforming growth factor-*β* (TGF*β*) signalling and changes in redox balance as pivotal effectors [94]. Both angiotensin-II and TGF*β* stimulate transformation of interstitial fibroblast into myofibroblasts by a NOX-dependent redox regulation [99, 100]. Angiotensin-II also increases activating protein-1 (AP-1) and related profibrotic genes, such as endothelin-1 [101]. ROS trigger latent TGF*β* and other profibrotic factors, such as connective tissue growth factor (CTGF) [102]. In addition, direct evidence implicating NOX in cardiac fibrosis comes from studies in genetically modified mice. NOX2-null mice showed significantly reduced interstitial fibrosis after RAAS activation [103]. Nevertheless, NOX and ROS can induce metalloproteinase (i.e., MMP9) expression, which degrades extracellular matrix and attenuates fibrosis [104, 105]. In this sense, activated PPAR*α* or PPAR*β*/*δ* by lipids can also interact with Smads and reduce the TGF*β* response (Figure 2(b)), suggesting PPAR-associated antifibrotic properties [106, 107]. 

### 4.4. ROS and Cardiac Cell Death

Although cell death scarcely occurs in the healthy myocardium (myocytes rarely proliferate in adult cardiac muscle), it is a feature of endstage heart failure. In the diabetic heart, the cell death-induced event is mainly represented by apoptosis and necrosis [49]. The former entails two major signalling pathways, extrinsic and intrinsic, with limited crosstalk between the two. Basically, in the extrinsic via, death ligands such as TNF*α* or Fas ligand bind to cognate receptors on the plasma membrane. These receptors contain an intracellular death domain, which recruits and activates caspase-8 through specific adaptors (i.e., TRADD or FADD (TNFR1-associated or Fas-associated death domain proteins, resp.)). Then, caspase-8 activates caspase-3, resulting in apoptosis. Interestingly, ROS overexpress TNF*α* by an apoptosis signal-regulating kinase-1- (ASK1-) NF*κ*B-dependent pathway [36]. Moreover, NF*κ*B can be involved in the upregulation of proapoptotic genes including FasL, Fas, c-Myc, and p53 [108–110], and ROS mediate downregulation of antiapoptotic factors like FLIP (FLICE/caspase-8 inhibitory protein) [111]. On the other hand, in type I diabetic hearts, ROS stimulate the epigenetic repression of cell cycle inducers, such as cyclin D1 [112]. 

In the intrinsic pathway, the oxidizing mitochondria are critical for the fate of the diabetic heart. A wide range of mitochondrial ROS-induced damages have been described in the mitochondrion, in particular, on its mPTP [2]. Oxidative modifications of proteins of the mPTP deregulate the mitochondrial anion fluxes allowing the entry of small solutes into the matrix [113]. Mitochondrial membrane potential is dissipated, ATP production declines, and matrix swells due to the movement of water from interstitial spaces (osmotic swelling) [114]. Cytochrome-C, which is tightly attached to the inner mitochondrial membrane by association with cardiolipin, is liberated to the cytosol to form the Apaf-1-procaspase-9 apoptosome complex that activates downstream effector caspases [115]. Nonscavenged ROS can also stimulate proapoptotic Bcl-2 family members, such as Bax and Bak [35]. Also, diverse components of the antioxidant defense and the complexes I-IV of the respiratory chain are closely exposed to ROS. The oxidation of these proteins decreases their enzymatic activity and disturbs the whole respiratory process [7, 116]. Mitochondria have a limited repair activity against DNA damage since they do not have complex chromatin organization, introns, or potent DNA-protecting enzymes [38]. Thus, ROS and lipid peroxides can also attack mtDNA. In the diabetic mitochondria, ROS modify the transcriptional activity of Tfam and thus decrease the expression of target genes such as cytochrome-B and ATP synthase subunit-6 [37, 38]. Furthermore, lipid peroxides and ∙OH^−^ may affect membrane fluidity/permeability by oxidizing MUFA/PUFA phospholipids [117]. In addition, diabetic mitochondria can be autophagied (a process named mitophagy) in a redox-dependent way. Mitophagy initially responds to a surviving mechanism, but, later, it consists of a lethal response alternative to apoptosis [118]. 

Finally, as with other cardiomyopathies, in DCM, necrosis occurs as an uncontrolled process involving a robust inflammatory component. Moderated quantities of ROS elicit cell apoptosis, but high amounts initiate cell necrosis. Diabetes leads to cardiac apoptosis before necrosis, and, when necrosis occurs, ventricular dysfunction follows [119]. Necrosis is a cell death lacking the characteristics of programmed cell death and characterized by early plasma membrane rupture and swelling of cytoplasmic organelles, in particular, mitochondria [120]. This rupture also results in the leakage of intracellular contents into the extracellular compartment with stimulation of inflammatory process and collagen accumulation. The intracellular Ca^2+^ overloading observed in diabetes induces intramitochondrial Ca^2+^ excess, which increases oxidative stress and mPTP opening and consequent osmotic swelling, organellar dysfunction, and necrosis [121]. Activation of the RAAS correlates with increased oxidative stress and necrosis in cardiomyocytes of diabetic patients [122]. Intriguingly, the coexistence of diabetes and hypertension increases necrosis, whereas there is no additional increase in apoptosis. Also, the resistance to IGF-1 observed in diabetes results in myocyte necrosis and myocardial dysfunction [123].

## 5. Conclusions

In the diabetic heart, several factors can trigger ROS production, especially, the excessive FA accumulation and metabolism. Unfortunately, natural ROS defenses cannot neutralize these deleterious substances and therefore leave the myocardium exposed to oxidative attacks on protein, lipids, and DNA of critical intracellular pathways and organelles, enhancing inflammatory, hypertrophic, fibrotic, and cell-death events in the diabetic myocardium. Antioxidant treatments have demonstrated beneficial effects in DCM pathology. However, ROS can also play key functions in the physiology of the cardiac diabetic scenario. Alternatively, exogenous stimulation of PPAR isoforms, such as PPAR*β*/*δ*, may facilitate lipid degradation and control of prooxidant mediators such as NOX and NF*κ*B.
